# Influence of vertical mucosal thickness and keratinized mucosal width on peri-implant health and marginal bone loss: a prospective study with a 2-year follow-up

**DOI:** 10.4317/medoral.26280

**Published:** 2023-11-22

**Authors:** Osman Babayiğit, Fatma Uçan-Yarkaç

**Affiliations:** 1 Department of Periodontology, Faculty of Dentistry, Necmettin Erbakan University, Konya, Turkey

## Abstract

**Background:**

Although it is known that the soft tissues around dental implants have an impact on its health and cause marginal bone loss, it is still uncertain exactly how. The aim of the study is to evaluate the effect of vertical mucosal thickness and keratinized mucosal width on marginal bone loss and periodontal clinical parameters in the 2-year follow-up of implants placed at the bone level.

**Material and Methods:**

87 bone-level dental implants were placed in 31 patients. The initial vertical mucosal thickness (VMT) was recorded at implant placement. At the second year follow-up, gingival index (GI), plaque index (PI), probing depth (PD), bleeding on probe (BOP), radiographic marginal bone loss (MBL) and width of the keratinized mucosa (KMW) were all measured. MBL and periodontal clinical parameters were evaluated separately according to VMT and KMW. VMT was categorized into two groups, Group 1 (≤ 2mm) and Group 2 (> 2 mm). KMW was divided into two groups, Group A (< 2mm) and Group B (≥ 2 mm).

**Results:**

Dental implants had a mean MBL of 0.39 ± 0.57 mm in the 2-year follow-up. MBL in Group 1 and 2 was 0.39 ± 0.42 mm and 0.38 ± 0.65 mm, respectively. MBL in Group A and B was 0.41 ± 0.68 mm and 0.37 ± 0.49 mm, respectively. No significant difference in MBL was found in the KMW and VMT groups (*p*>0.05). The group with the thicker vertical mucosa was shown to have statistically substantially higher PI and GI values (*p*=0.040 and *p*=0.014, respectively).

**Conclusions:**

Within the limits of the present study, it was observed that the vertical mucosal thickness and the width of the keratinized mucosa did not affect the marginal bone loss. In addition, it was observed that the insufficiency of the width of the keratinized mucosa did not affect the periodontal clinical parameters, but the thicker vertical mucosa could increase the plaque index and gingival index.

** Key words:**Alveolar bone loss, bone-implant interface, dental implants, dental plaque index, phenotype, tissues.

## Introduction

Dental implants are titanium-based synthetic tooth roots that are placed in the jawbone to support dentures, bridges, and other types of dental restorations. One of the most crucial elements for the long-term success of dental implants is the stability of the surrounding bone and soft tissues (1). Marginal bone loss (MBL) is a natural process that occurs after the placement of a dental implant, but excessive MBL can lead to implant failure. Studies have shown that the amount of MBL around dental implants varies depending on several factors such as implant design, implant surface, surgical technique, infection, occlusal overload, biomechanical factors and the patient's oral hygiene (2,3).

The morphological and physical characteristics that describe the clinical appearance of the tissues encircling and supporting osseointegrated implants are known as the peri-implant phenotype (4). The peri-implant phenotype consists of a bone component with peri-implant bone thickness and a soft tissue component with peri-implant keratinized mucosa width (KMW), vertical and horizontal mucosal thickness (5). This concept covers the lingual and palatal peri-implant regions in addition to the buccal regions. The peri-implant phenotype is specific to a location, just as the periodontal phenotype, and it can alter over time in response to changes in the environment. According to a recent literature review, bone remodeling around dental implants may be influenced by the phenotype of peri-implant mucosa, which comprises horizontal and vertical mucosal thickness (6,7).

A particular volume of soft tissue is necessary to create a biological width around dental implants, much like teeth do, according to research conducted on animals by Berglundh and Lindhe (8). To determine the effect of mucosal thickness around implants, Suárez-López Del Amo *et al*. performed a meta-analysis of five 12-month short-term studies (2). According to this investigation, thicker mucosa (>2 mm) surrounding implants at the outset encouraged the development of biologic width and decreased the rate of short-term marginal bone loss (2).

According to clinical research, implants placed into mucosal tissues that 2 mm or thicker have a considerably lower risk of causing bone remodeling and bone loss (2). In addition, the association between tissue thickness and crest bone loss has also been supported by a large number of investigations from various research institutions (9). Summarizing the data from these studies, a systematic review was published concluding that initial placement of dental implants in a thinner vertical mucosa had greater radiographic MBL at short-term follow-up (10). A more recent systematic review, however, failed to support the advantage of thicker tissue over thin tissue in preserving bone integrity (10,11).

The majority of research indicates that peri-implant diseases are more likely to occur in individuals with thin horizontal and vertical mucosa, as well as a deficiency of keratinized mucosa (12). Some studies have shown that implants lacking keratinized mucosa are associated with plaque deposition, mucosa inflammation, regression, and loss of attachment, although not with radiographic bone loss (13). However, research evaluating the impact of mucosal thickness on the long-term survival of implants and the health of peri-implant tissue is scarce.

Therefore, the aim of our study is to evaluate the influence of keratinized mucosal width and vertical mucosal thickness on marginal bone loss in the 2-year follow-up of implants placed at the bone level.

## Material and Methods

- Study design and patient selection

This study included 31 patients who applied to the Department of Periodontology, Faculty of Dentistry, Necmettin Erbakan University between February 2019 and March 2020 for dental implant treatment due to edentulousness. Standardized intra-oral periapical radiographs of 31 patients with 87 dental implants that placed maxillary and mandibular posterior region, preoperatively and 2 years after the prosthetic loading, were analyzed.

This clinical prospective cohort study was conducted in agreement with the Declaration of Helsinki, following STROBE guidelines for cohort studies. The protocol for this study was approved by Necmettin Erbakan University Faculty of Dentistry Non-Pharmaceutical and Medical Device Research Ethics Committee (ref. 2021/13-98) for studies involving human subjects. All the selected patients gave their informed consent to participate in this study.

The inclusion criteria for enrollment in this study were as follows.

Age >18 years;

Healthy patients without any uncontrolled systemic disease;

Patients who want to have dental implants in the area with missing teeth;

Complete digital panoramic radiographs obtained preoperatively and intra-oral periapical radiographs obtained 2 years after prosthetic loading;

Obtaining sufficient primary stability during implant placement (≥20 N/cm)

The exclusion criteria were as follows:

Patients using regular medications that may affect wound healing;

Smoking more than 10 cigarettes a day;

Patients with history of periodontitis;

Patients who had poor oral hygiene;

Patients with diseases affecting bone quality (osteoporosis, e.g.)

Use of bone graft and membrane materials in surgical procedures;

Technical radiographic artifacts interfering with analysis.

**Figure 1:**
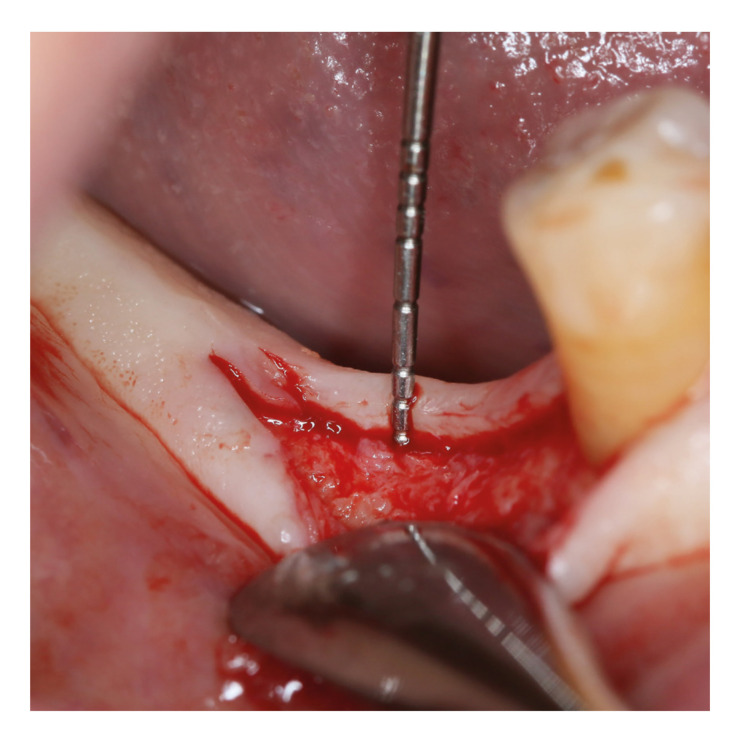
Measurement of vertical mucosal thickness.

All surgical interventions were performed by the same surgeon (F.U.Y.). Prior to the operation, the patients rinsed with 0.2% chlorhexidine (Klorhex, Drogsan, Ankara, Turkey) mouthwash for 1 min. After infiltrative local anesthesia (Ultracaine DS; Hoechst), full‐thickness buccal flap was elevated, taking care to preserve the keratinized mucosa, and lingual flap was not elevated. At the bone crest in the area designated for the placement of the future implant, VMT was measured using a 1.0 mm marked periodontal probe (Hu Friedy; Chicago, IL, USA) (Fig. 1) (10). VMT was divided into groups as thin if it was 2 mm or less (Group 1) and thick if it was more than 2 mm (Group 2) (10). The implant site was prepared 1.5 mm from the adjacent teeth and 3 mm from the adjacent implant, leaving at least 1 mm of bone in the buccal and lingual. Implants with the same macrogeometric shape were placed at the bone level with a 2-stage approach. After implant insertion mucoperiosteal flaps were repositioned with tension-free sutures. The patients received oral and written oral hygiene instructions following surgery.

Patients who had dental implants were called again after 2 years. Periodontal parameters such as gingival index (GI), plaque index (PI), probing depth (PD), and bleeding on probing (BOP) were measured and recorded (14). Also, the width of the keratinized mucosa and radiographic marginal bone loss (MBL) around the implants were measured.

**Figure 2:**
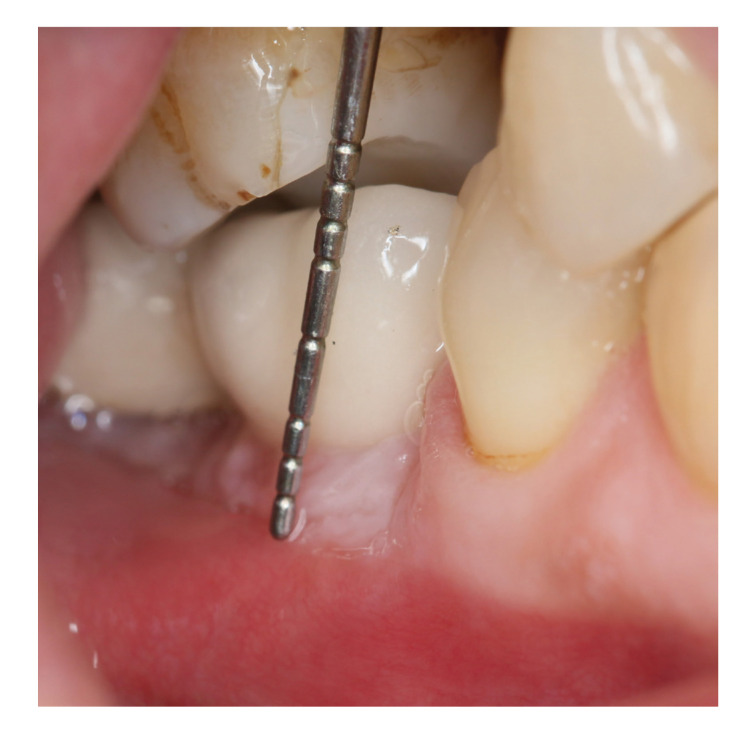
Measurement of keratinized mucosal width.

Keratinized mucosal width is defined as the distance from the free gingival border to the mucogingival junction. Using a periodontal probe, this value was measured as the distance between the mucogingival margin and the gingival border at the implant's most apical position to the nearest 1 mm (Fig. 2) (15).

**Figure 3:**
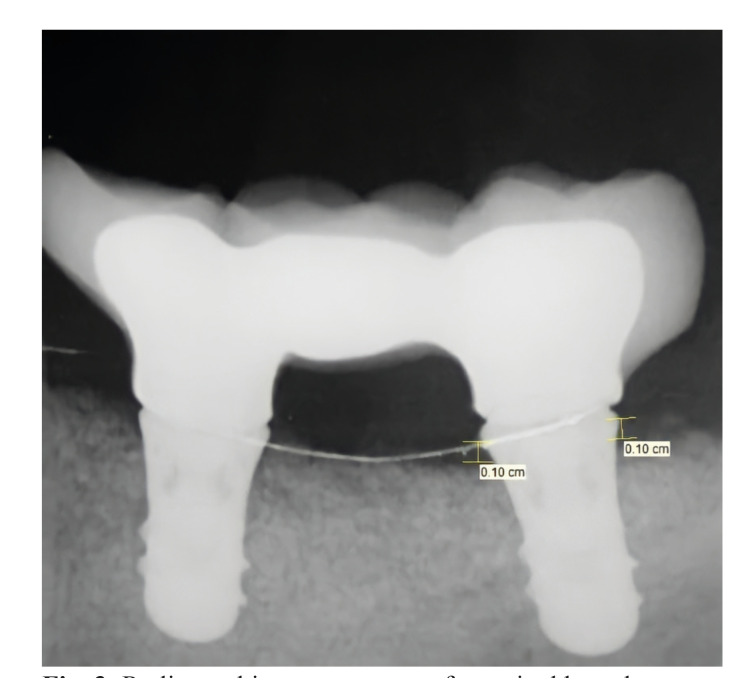
Radiographic measurement of marginal bone loss.

Intraoral periapical radiographs were taken 2 years after prosthetic loading to measure marginal bone loss of each implant. Radiographs were taken in a way that made it possible to see the threads and the implant-abutment interface clearly. On the distal and mesial sides of each implant, the distances between the implant shoulder and the initial bone to implant contact were measured (Fig. 3). The known implant length and diameter were both used to calibrate the measurements. The highest bone loss measured from the distal and mesial was recorded.

Radiographic MBL measurements were performed by a blinded examiner (O.B.) using a software program (Turcasoft Dent, Samsun, Turkey). For better visualization, the radiographs were magnified and seen in full screen mode. The measurements were calibrated using the known implant diameter and length.

- Statistical analysis

The power analysis was 80%, with a test significance threshold (α) of 5%. A computer algorithm with two-sided equivalency for the difference of proportions in the two group design was used to determine the sample size for each group (nQuery Advisor, version 7.0; Statsols, Los Angeles, CA.). SPSS 23.0 (Software by IBM SPSS Statistics for Windows, Version 23.0, Armonk, NY: IBM Corp) was used to analyze statistical data. The data were presented with mean values and standard deviations (±). The Kolmogorov-Smirnov test revealed that the data were distributed normally. For data that weren't distributed regularly, a non-parametric test was used. Categorical variables were subjected to the Chi-square test. Using the Mann-Withney U test, two groups were compared. Spearman correlation (rho) coefficients were used to assess the relationships between the variables. Using a 95% confidence level, differences were considered statistically significant when *p*<0.05.

## Results

31 patients (18 females and 13 males) with a mean age of 47 years (range: 21-69 years) who were followed up prospectively for 2 years were included in the study. A total of 87 bone level implants were placed in 44 female patients and 43 in male patients. The mean age of the female patients was 43 years, and that of the male patients was 53 years. Table 1 displays the demographic data of the patients and the characteristics of the implants placed in both VMT groups.

**Table 1:**
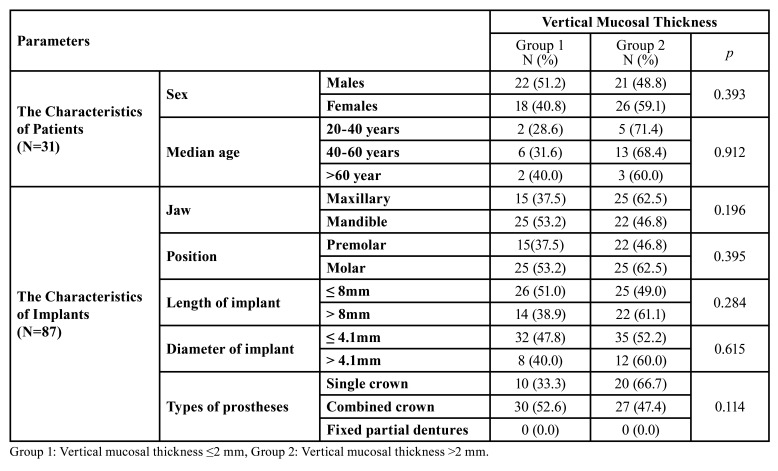
Characteristics of patients and implants.

Dental implants had a mean marginal bone loss of 0.39 ± 0.57 mm, with a range of 0 to 3.5 mm. The mean keratinized mucosal width was 2.22 ± 1.46 mm, ranging from 0 to 7 mm. The mean vertical mucosal width was 2.57 ± 0.86 mm, ranging from 1 to 5 mm. The mean PI, GI, BOP and PD values were 0.82 ± 0.40, 0.33 ± 0.49, 0.73 ± 0.44 and 3.40 ± 0.72 mm, respectively.

When the correlation of the data in Table 2 was examined, a statistically significant difference was observed between the vertical mucosal thickness and plaque index values according to the KMW (*p*<0.05). It was observed that as the KMW increased, the vertical mucosal thickness and plaque index also increased (*p*<0.05). In addition, statistical significance was observed between pocket depth and marginal bone loss (*p*<0.05). MBL is exacerbated by increasing pocket depth (*p*=0.007). GI, PI, BOP and PD data all showed a statistically significant positive link (*p*<0.05).

Two categories of initial vertical mucosal thicknesses and keratinized mucosa widths were identified around dental implants. Group 1 included those with a vertical mucosal thickness of 2 mm or less, and Group 2 included those with a vertical mucosal thickness greater than 2 mm. Group A refers to keratinized mucosa widths of 2 mm or less, and Group B refers to those above 2 mm.

Dental implants were distributed based on the VMT and the KMW. Group 1 (VMT ≤2 mm) had a total of 40 implants, 23 dental implants with a KMW of 2 mm or greater, and 17 implants with a KMW of less than 2 mm. Group 2 (VMT >2 mm) had a total of 47 implants, 28 dental implants with a KMW of 2 mm or greater, and 19 implants with a KMW of less than 2 mm.

The comparisons of MBL, GI, PI, BOP, and PD between the two VMT groups are shown in Table 3. While there was no statistically significant difference between MBL, BOP and PD values between VMT groups (*p*>0.05), statistically significant differences were found in PI and GI values (*p*<0.05). PI and GI values were found to be statistically significantly higher in the group with a VMT of 2 mm or less (*p*<0.05). This can be clarified through the idea that a thin VMT promotes greater plaque accumulation and increases the susceptibility of the gingival defense line to inflammation.

**Table 2:**
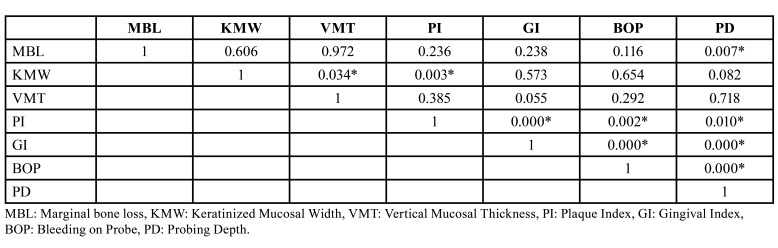
Correlation of data.

**Table 3:**
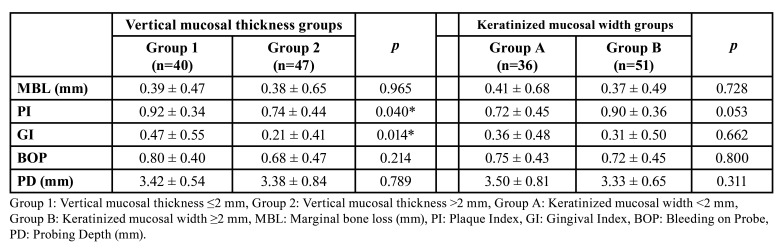
Comparison of MBL, PI, GI, BOP and PD values based on vertical mucosal thickness and keratinized mucosal width groups.

The findings from the comparisons of the MBL, GI, PI, BOP, and PD between the two KMW groups are presented in Table 3. No statistically significant difference was found between the two KMW groups in any of the compared data (*p*>0.05).

The correlation of MBL values in the VMT and KMW groups with each other was evaluated (Table 4). No statistically significant difference was found in the comparison of marginal bone loss in the Group A according to thin and thick vertical mucosal thickness (*p*>0.05). Likewise, there was no statistically significant difference in the comparison of marginal bone loss in the Group B in terms of thin and thick vertical mucosal thickness (*p*>0.05).

**Table 4:**
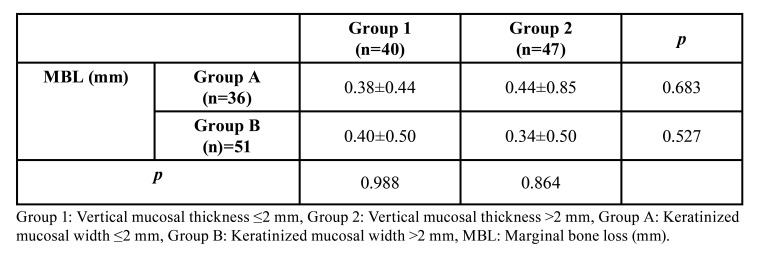
Correlation of VMT and KMW groups with each other according to MBL (mm).

## Discussion

The aim of our study was to evaluate periodontal clinical parameters and marginal bone changes around implants in relation to keratinized mucosal width and initial vertical mucosal thickness. Cone-beam computed tomography (CBCT), biometric scanner use, color probe translucency method, non-ionized ultrasonography or transmucosal measurement methods with an endodontic spreader have been shown to measure mucosal thickness in the literature (16). In our study, a periodontal probe was used after partial flap elevation during implant surgery stages to measure initial vertical soft tissue thickness. This is also the most common technique known.

Accurate measurement of peri-implant marginal bone loss is difficult. The most reliable results are obtained using histology techniques, however they cannot be applied to dental implants without complications (17). Although cone-beam computed tomography provides more accurate information due to its 3-dimensional image, studies have shown that there is no significant difference in measurement error between parallel intraoral radiographs and CBCT. Also, given the "as low as reasonably achievable" (ALARA) principle, it was decided to use parallel intraoral radiographs for marginal bone loss measurements in this study (18).

Marginal bone loss and peri-implant diseases are still the most researched and not yet clarified topics in oral implantology. The importance of vertical mucosal thickness was recently discovered and its effects on bone loss are still being investigated. Recent studies in the literature accept a threshold value of 2 mm in vertical mucosal thickness (2). Linkevicius *et al*. compared the marginal bone loss of 40 implants with a VMT of 2 mm or less and 40 implants with a VMT of more than 2 mm. MBL values measured after 1 year of prosthetic loading were statistically significantly higher in the low VMT group (*p*< 0.001) (19). Likewise, Van Eekereen *et al*. also showed that there was statistically significantly less marginal bone loss in the bone level implants group with a VMT greater than 2 mm (17). The results of our study contradict with this study. By classifying the 87 bone level implants into 2 groups based on VMT, MBL in the second year of follow-up was assessed. After two years of loading, the dental implants in Group 1 (≤2 mm of initial VMT) exhibited a MBL of 0.39 mm, and the implants in Group 2 (>2 mm of initial VMT) showed a MBL of 0.38 mm.

Only a limited number of studies have examined the relationship between vertical mucosal thickness and peri-implant health, aside from those that center on marginal bone loss (20). These limited studies have stated that thick VMT may increase MBL and PD in patients with a history of periodontitis (21). Zhange *et al*. found that the risk of peri-implantitis increased 1.5 times for every 1 mm of vertical mucosal thickness increase in patients with a history of periodontitis and emphasized that excessive soft tissue thickness may have negative effects on peri-implant health (20). In the results of our study, it was observed that PI and GI scores increased statistically as the vertical mucosal thickness decreased (*p*<0.05). This can be explained by the fact that, unlike the study of Zhang *et al*., patients with a history of periodontitis were not included in our study.

Peri-implant keratinized mucosa is a defensive layer that protects the dental implant against external factors. The importance of keratinized mucosa in maintaining oral hygiene has been shown in many studies (22-24). There may be reductions in the width of the keratinized mucosa due to reasons such as gingival recession, tooth extraction, periodontal abscesses or peri-implantitis. Studies in the literature show that 2 mm of keratinized mucosa around the tooth or dental implant is sufficient to prevent plaque accumulation and to ensure the health and stability of the peri-implant tissues (25,26). However, other investigations found no appreciable change in plaque index score with keratinized mucosa present or absent (15,27). Some studies have shown that the risk of bleeding at implant sites with narrow keratinized mucosa (less than 2 mm) was considerably higher than the risk at sites with wide keratinized mucosa (more than 2 mm) (25,26,28). However, other investigations shown that neither the GI nor the mucosa's propensity to bleed were affected by the KMW (27,29). In our study, no statistically significant relationship was found between the KMW and periodontal clinical parameters. This finding would suggest that patients with insufficient KMW can prevent periodontal diseases by providing oral hygiene care correctly.

The link between KMW and MBL has been the subject of numerous investigations, however there is still controversy about this subject (15,25,26,30). Bouri *et al*. conducted a study in which they compared KMW with MBL and periodontal clinical parameters of 200 dental implants (76 patients) of 4.5 years. The MBL was 1.24 mm in the group with wide KMW, compared to 1.72 mm in the group with narrow KMW. The study's findings demonstrated statistically that less than 2 mm of KMW promotes higher MBL, PI score, GI score, and bleeding on probing.

The MBL was assessed in 100 patients with 276 dental implants in a 1-year follow-up research by Kim *et al* (15). As a result, the group with narrow KMW had a 0.65 mm bone loss, whereas the group with wide KMW had a 0.41 mm bone loss. The outcomes were statistically significant. In a study by Adibrad *et al*., they followed a 2-year follow-up of 66 dental implants applied to 27 patients (25). They found MBLs of 1.12 mm and 1.24 mm in the wide KMW group and narrow KMW group, respectively. As seen by their findings, there was no statistically significant difference between the two groups. In an 8-year follow-up study by Chung *et al*., they evaluated MBL according to KMW of 339 dental implants applied to 69 patients (30). As a result, no significant difference was found between the KMW groups in terms of MBL (0.11 mm MBL in both groups). In our research, there was no MBL difference between the KMW groups. When KMW and VMT values were compared in terms of their impact on MBL, no statistically significant difference was found (*p*>0.05).

## Conclusions

In the current literature, there are conflicting findings about the influence of keratinized mucosa on MBL and peri-implant health. Within the limits of the present study, it was observed that the vertical mucosal thickness and the width of the keratinized mucosa did not affect the MBL. In addition, it was shown that the insufficiency of the KMW did not affect the periodontal clinical parameters, but the thicker vertical mucosa could increase the PI and GI. The findings from this clinical study need to be confirmed by further prospective studies with larger sample sizes and longer follow-up.
